# Gastric volvulus in an elderly male with a hiatal hernia and a wandering spleen: a case report

**DOI:** 10.1093/jscr/rjaf808

**Published:** 2025-10-16

**Authors:** Faris Alsobyani, Renad AbuAlshamat, Ghadeer Alharthi, Samah Khayyat, Ahmed Gadah

**Affiliations:** Department of General Surgery, Al Noor Specialist Hospital, 3rd Ring Road, 24241 Makkah, Saudi Arabia; Department of General Surgery, Al Noor Specialist Hospital, 3rd Ring Road, 24241 Makkah, Saudi Arabia; Department of General Surgery, Al Noor Specialist Hospital, 3rd Ring Road, 24241 Makkah, Saudi Arabia; Department of General Surgery, Al Noor Specialist Hospital, 3rd Ring Road, 24241 Makkah, Saudi Arabia; Department of General Surgery, Al Noor Specialist Hospital, 3rd Ring Road, 24241 Makkah, Saudi Arabia

**Keywords:** gastric volvulus, mesenteroaxial rotation, hiatal hernia, wandering spleen, gastropexy

## Abstract

Gastric volvulus is a rare surgical emergency that can lead to obstruction, ischemia, and necrosis if not promptly managed. We report a unique case of a 78-year-old male who presented during the 2024 Islamic pilgrimage season with symptoms of epigastric pain and persistent vomiting. Imaging revealed a mesenteroaxial gastric volvulus associated with a large hiatal hernia and a wandering spleen—an uncommon triad, especially in adults. The patient underwent successful laparoscopic reduction of the volvulus, cruroplasty, Toupet fundoplication, and anterior gastropexy. Postoperative recovery was uneventful, and the patient was discharged in good condition. This case highlights the importance of high clinical suspicion, appropriate imaging, and timely surgical intervention in managing gastric volvulus. It also underscores the need to identify and address contributing anatomical abnormalities to prevent recurrence. To our knowledge, this is the first reported adult case of concurrent gastric volvulus, hiatal hernia, and wandering spleen.

## Introduction

Gastric volvulus is a life-threatening condition, defined as the abnormal twisting of the stomach, which could ultimately lead to obstruction, strangulation, and necrosis [1]. During the Islamic pilgrimage season of 2024, we were presented with a unique case of gastric volvulus that occurred simultaneously with a hiatal hernia and a wandering spleen. In this case report, we present the unique findings and discuss the current literature regarding this rare occurrence.

## Case report

We were presented with a 78-year-old male pilgrim during the 2024 Islamic pilgrimage season. The patient is a known case of valvular heart disease with pulmonary hypertension, presented to our emergency department complaining of epigastric and left upper quadrant abdominal pain for the past 4 days. He was in his usual state until the sudden onset of the pain, with no radiation or shifting. It was associated with vomiting gastric contents around three to five times a day without any history of gastrointestinal (GI) bleeding. He denied having any fever, change in bowel habits, or abdominal distention. He reports maintaining his body weight without any change in appetite, night sweats, or significant fatigue. Regarding his surgical history, he had a prostate-related surgery >5 years ago, the details of which could not be recalled by the patient. The patient has no history of malignancy in the family.

**Figure 1:**
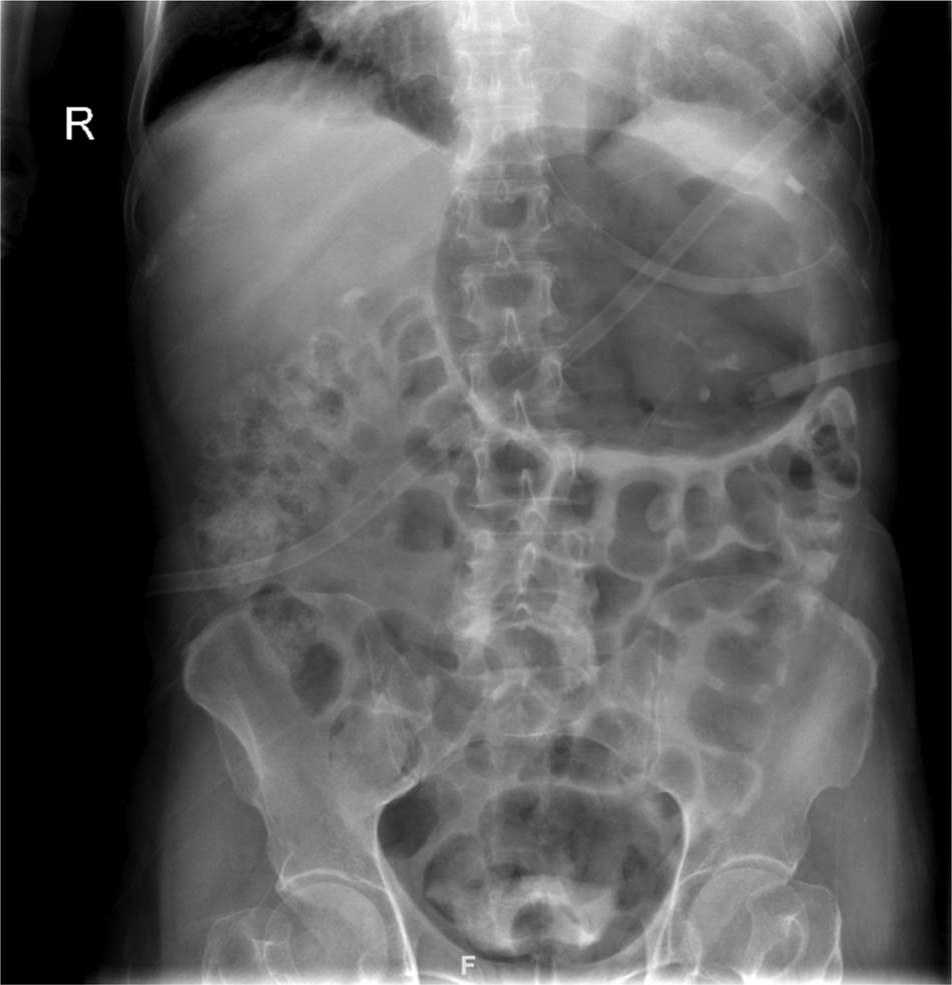
Abdominal radiograph in an anteroposterior (AP) projection demonstrates multiple dilated loops of small bowel with prominent air-fluid levels. The large bowel appears decompressed. A nasogastric tube is seen *in situ*, terminating in the stomach. No evidence of free intraperitoneal air is identified to suggest perforation. No radiopaque foreign bodies or mass lesions are visible.

**Figure 2:**
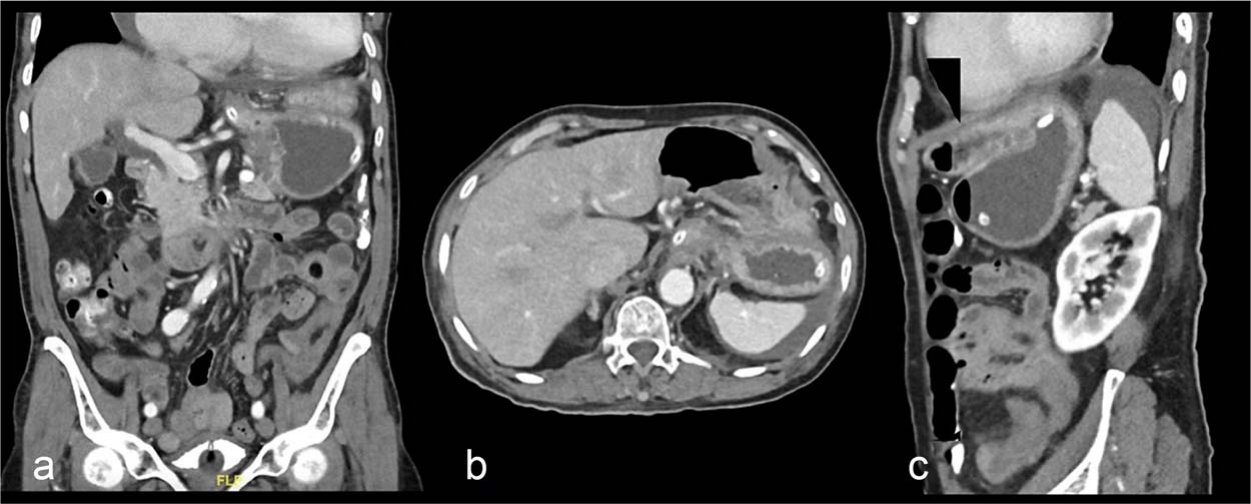
Contrast-enhanced CT abdomen and pelvis shows nasogastric (NG) tube noted in position. The stomach demonstrates abnormal orientation with inferior displacement of the fundus and superior displacement of the gastric pylorus near the gastroesophageal junction, with interval improvement of the gastric distension, likely mesenteroaxial gastric volvulus. Persistent mild perigastric and perisplenic fluid. Preserved gastric wall enhancement. Mild pelvic free fluid is noted. No free air or size significant lymph node.

On presentation, the patient was fully oriented with vitals in the normal range. On examination, the abdomen was soft and not distended. There was a scar at the lower midline laparotomy for an unknown indication. There was no abdominal tenderness or any signs of peritonitis. All hernial orifices were intact. His laboratory results were unremarkable with a normal renal and electrolytes profile and no leukocytosis or anemia. Abdominal radiograph demonstrates multiple dilated loops of small bowel with prominent air-fluid levels. No evidence of free intraperitoneal air is identified to suggest perforation. No radiopaque foreign bodies or mass lesions are visible (Fig. 1). The patient underwent computed tomography (CT) of abdomen with IV contrast. The stomach demonstrates abnormal orientation with inferior displacement of the fundus and superior displacement of the gastric pylorus near the gastroesophageal junction, with gastric distension. Mild perigastric and perisplenic fluid. Preserved gastric wall enhancement. Mild pelvic fluid is noted (Fig. 2).

**Figure 3:**
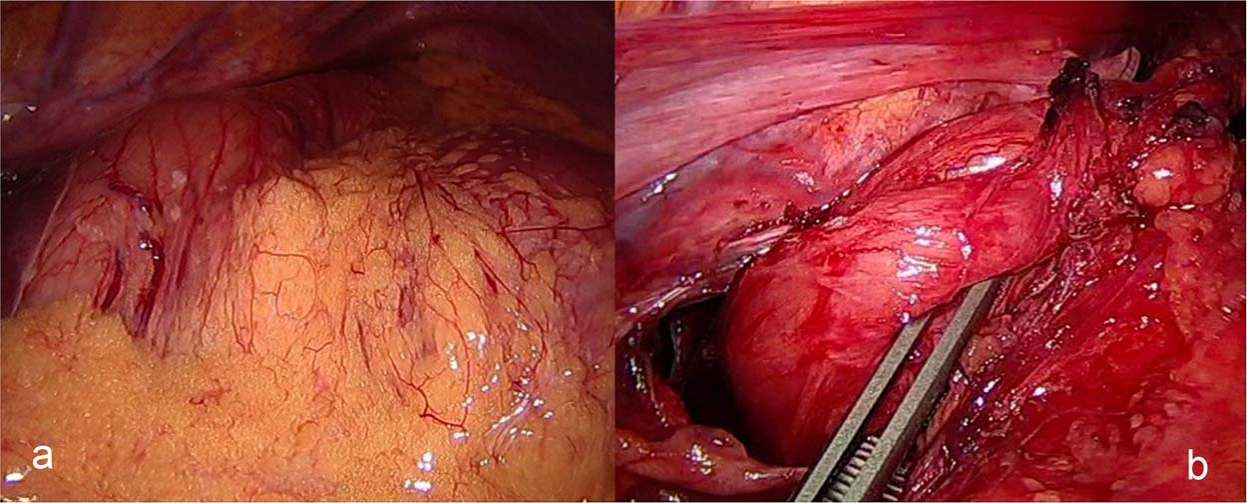
(a) Intraoperative image revealing the position of the stomach without any devitalized tissue. (b) A large hiatal hernia after completion of dissection in preparation for cruroplasty with a Toupet fundoplication.

**Figure 4:**
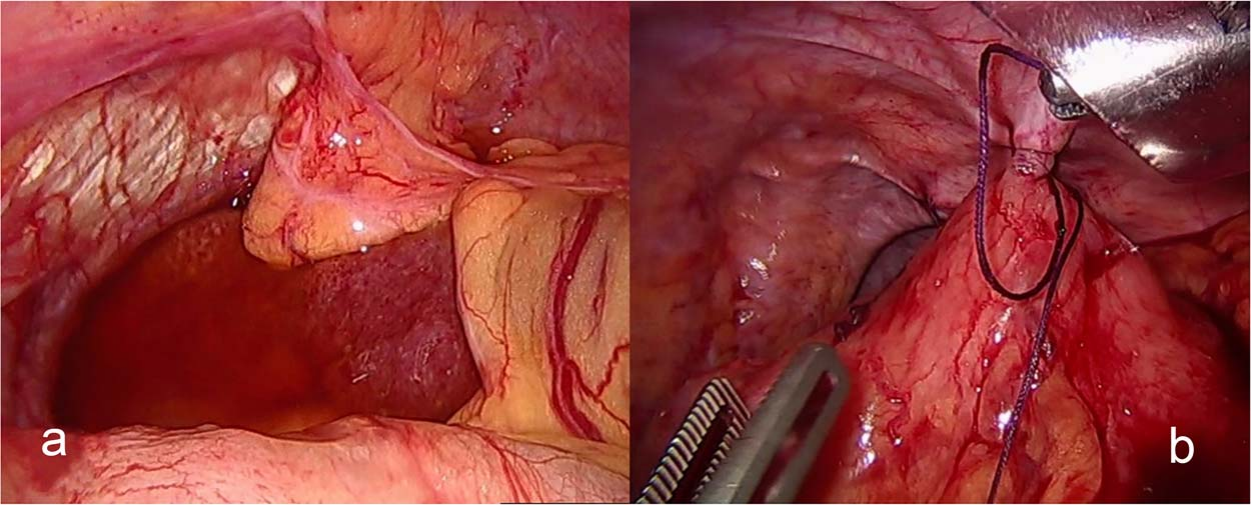
(a) The spleen appears healthy and in an abnormally mobile position, consistent with a wandering spleen, with absent or lax ligamentous attachments. (b) Postreduction view of the stomach, with its body sutured to the lateral abdominal wall (gastropexy) to prevent recurrence of mesenteroaxial gastric volvulus.

The patient was admitted under the care of the general surgery department, and he was taken to the operating room for a laparoscopic exploration, which showed mesenteroaxial gastric volvulus, wandering spleen, and a large hiatal hernia which was ~5 cm in size. No devitalized tissues were found. An attempt at intraoperative endoscopy failed to pass through the hiatus. A cruroplasty with a Toupet fundoplication and gastropexy were done as shown in (Figs 3 and 4). On the third postoperative day, the patient was doing well. He was tolerating oral intake and passing regular motions and was discharged home in good condition as he was traveling to his country.

## Discussion

Gastric volvulus is a medical emergency and can be defined as the abnormal rotation of the stomach >180°, which leads to a closed-loop obstruction that may result in strangulation and necrosis of the stomach [1]. There are primarily two types of gastric volvulus: organo-axial and mesentero-axial. Organo-axial volvulus is the twist of the stomach in line with the axis joining the gastroesophageal junction and the pylorus [2]. The other type of volvulus, i.e. less frequent in occurrence, is the mesentero-axial which involves rotation around an axis, i.e. located in the midline of the lesser and also the greater curvatures of the stomach [3].

Patients with gastric volvulus will present in what is known as the Borchardt triad, consisting of intense epigastric pain, intractable retching, and inability to pass a nasogastric tube. The chronic volvulus often presents with nonspecific features and in ill-appearing patients may present with symptoms of epigastric discomfort, abdominal distention, and a feeling of fullness even when consuming small amounts of food [4]. Due to the nature of symptoms, diagnosis is usually delayed, which worsens the condition and results in high risks of complications.

Various risk factors are implicated in the development of gastric volvulus, including anatomical factors like abnormal gastric ligaments, diaphragmatic hernias, and previous gastric surgeries [5]. Other conditions such as wandering spleen, which share a similar etiology of having abnormal intraperitoneal visceral ligaments, and hiatal hernias are particularly related to higher incidences of gastric volvulus [5]. Wandering spleen is particularly relevant as its mobility can contribute to the abnormal rotation of the stomach due to deficient fixation from abnormal ligamentous connections and a mobile spleen [6]. Similarly, hiatal hernias, characterized by the herniation of the stomach organs through the diaphragm, can modify the morphological arrangement of the stomach and relative structures and raise the risk of volvulus [7].

The prognosis of gastric volvulus depends on the time of diagnosis and treatment. Thus, acute gastric volvulus is a surgical emergency, because gastric ischemia and necrosis develop rapidly [8]. Chronic cases can often be managed conservatively but may require surgery if symptomatic. The main objective of the treatment is to avoid its relapse and restore the correct anatomical orientation of the stomach [1].

Diagnostic imaging is instrumental in diagnosing and managing gastric volvulus [9]. Abdominal plain radiographs may demonstrate signs of distal obstruction, such as a double-bubble or a large, distended gas-filled stomach; however, CT scans are more beneficial to establish the degree of volvulus and to exclude other causes like hiatal hernia or wandering spleen. In this case, the abdominal CT was very relevant in the evaluation and management of the patient since it was shown from the findings that it helped in diagnosing the condition.

Endoscopy can be diagnostic and interventional in the treatment of gastric volvulus. It enables direct visualization of the midline twisted stomach and can be attempted detorsion as was done in this particular case at the initial stage [1]. Even though the endoscopic attempts at detorsion failed, the endoscopy served to indicate the severity of the obstruction and its characteristics that suggested the need for surgical management. In less severe cases, endoscopic detorsion is feasible which helps untwist the volvulus without surgery, which is an important reason for its inclusion in the management plan.

Management measures will vary depending on the type and severity of the volvulus. Acute cases generally necessitate emergency surgery to decompress the stomach, untwist, and repair complications and any predisposing factors to prevent recurrence and perform gastropexy [9]. Although in chronic cases, a trial of conservative management with diet modification and postural changes as well as prokinetics and antisecretory medications can be attempted with a high success rate [9]. A splenopexy should be performed in a wandering spleen to prevent torsion to the splenic vascular pedicle [5].

There have been very few documented cases describing gastric volvulus in association with a wandering spleen; however, the vast majority were in pediatric patients. To our knowledge, no case has been reported with a similar occurrence in an adult patient with a hiatal hernia, wandering spleen, and gastric volvulus simultaneously.

## Conclusion

Gastric volvulus is a rare but serious condition where the stomach twists abnormally, causing severe pain, retching, and obstruction. It is diagnosed with imaging like CT scans and managed with emergency surgery for acute cases or conservative measures for chronic cases. Identification of associated conditions is crucial to prevent recurrence or future complications. Prompt diagnosis is crucial to prevent complications like tissue damage and necrosis.
